# Sacubitril/Valsartan for Prevention of Cancer Therapy-Related Cardiac Dysfunction: A Systematic Review and Meta-Analysis of Randomized Controlled Trials

**DOI:** 10.3390/jcdd13070323

**Published:** 2026-07-10

**Authors:** Hugo Lopez-Arevalo, Hans Mautong, Adrian Nubla, Marco Antonio Dispagna, Ninos Nissan

**Affiliations:** 1 Department of Hospitalist Medicine, Merrimack Health Lawrence Hospital, Lawrence, MA 01841, USA; 2 Department of Internal Medicine, John H. Stroger, Jr. Hospital of Cook County, Chicago, IL 60612, USA; 3 School of Health, Universidad Espíritu Santo-Ecuador, Samborondón, Guayas 60612, Ecuador; 4 Division of Cardiology, University of Illinois, Chicago, IL 60612, USA

**Keywords:** sacubitril/valsartan, cardiotoxicity, anthracyclines, global longitudinal strain, cancer therapy-related cardiac dysfunction, meta-analysis

## Abstract

Cancer therapy-related cardiac dysfunction (CTRCD) is a significant complication of anthracycline-based regimens and anti-HER2 agents. Global longitudinal strain (GLS) detects subclinical dysfunction before ejection fraction decline and is recommended by the 2022 ESC guidelines for surveillance. Sacubitril/valsartan, an angiotensin receptor–neprilysin inhibitor, has shown cardioprotective potential in recent trials. Following PRISMA 2020 guidelines, we searched PubMed, EMBASE, and Cochrane CENTRAL through February 2026. Three RCTs met eligibility criteria (*n*= 350; PROSPERO: CRD420261383124): Hsu 2025 (*n* = 100, 12 months), PRADA II 2025 (*n* = 138, 18 months), and SARAH 2025 (*n* = 112, 6 months). Random-effects meta-analysis used REML with Hartung–Knapp–Sidik–Jonkman adjustment. The pooled mean difference in final GLS significantly favored sacubitril/valsartan (MD −0.95%; 95% CI −1.40 to −0.50; *p* = 0.012; I^2^ = 0%). Final LVEF showed a consistent trend (MD +1.53%; 95% CI −0.47 to 3.52; *p* = 0.082; I^2^ = 0%). Hypotension was numerically more frequent with sacubitril/valsartan (OR 5.10; 95% CI 0.52–49.50; *p* = 0.091). Sacubitril/valsartan initiated during anthracycline therapy was associated with significant GLS preservation. However, GLS is a surrogate imaging marker and hard clinical events were rare or absent, so the modest effect magnitude and limited number of trials warrant cautious interpretation; the clinical benefit remains uncertain and requires confirmation in adequately powered trials with hard clinical endpoints.

## 1. Introduction

Contemporary cancer therapy frequently combines anthracyclines with other potentially cardiotoxic agents, including taxanes, human epidermal growth factor receptor 2 (HER2)-targeted therapies such as trastuzumab, and thoracic radiation, creating an additive or synergistic risk for cardiac injury [1,2,3]. Anthracycline-associated cardiotoxicity is dose-dependent and can progress to irreversible heart failure, while trastuzumab causes a distinct, generally reversible form of myocardial dysfunction that is amplified by prior anthracycline exposure [1,3]. In a prospective cohort of 2625 anthracycline-treated patients, the overall incidence of CTRCD was 9%, with 98% of events occurring within the first year after treatment [4]. When subclinical dysfunction is captured using more sensitive measures such as global longitudinal strain (GLS), the incidence ranges from 9% to 44% across studies [5].

Moreover, echocardiographic GLS by speckle-tracking imaging has emerged as a more sensitive, reproducible, and preferred marker of early myocardial dysfunction than left ventricular ejection fraction (LVEF) [6]. A meta-analysis of 21 prognostic studies demonstrated that a relative decline in GLS during chemotherapy was associated with a 16-fold higher risk of subsequent CTRCD (OR 15.82; 95% CI 5.84–42.85) [5]. The current available evidence has led the 2022 European Society of Cardiology (ESC) guidelines on cardio-oncology to incorporate GLS into the formal diagnostic criteria for CTRCD, defining a relative decline of ≥15% as clinically significant subclinical dysfunction [7]. Beyond echocardiography, multimodality cardiovascular imaging—including cardiac magnetic resonance and cardiac computed tomography—is increasingly used for the detection and characterization of cancer therapy-related cardiac injury [8,9].

Neurohormonal blockade with angiotensin-converting enzyme (ACE) inhibitors, angiotensin receptor blockers (ARBs), and beta-blockers has been the main pharmacologic strategy for cardioprotection during anthracycline therapy. However, randomized trials of these agents have yielded inconsistent results, with modest or no significant effects on LVEF preservation [10,11,12,13,14]. Recent reviews have concluded that the optimal cardioprotective strategy for anthracycline-treated patients remains undefined [15,16].

Recently, attention has shifted towards sacubitril/valsartan, an angiotensin receptor–neprilysin inhibitor (ARNI) that offers dual neurohormonal blockade through simultaneous inhibition of the renin–angiotensin system and augmentation of natriuretic peptide signaling [17]. Preclinical studies have demonstrated that sacubitril/valsartan attenuates doxorubicin-induced systolic dysfunction, reduces oxidative stress, and inhibits myocardial fibrosis and inflammation through pathways that include AMPKα-mTORC1 signaling [18,19].

Three recent RCTs—Hsu et al. [20], PRADA II [21], and the SARAH trial [22]—have independently evaluated sacubitril/valsartan for prevention of CTRCD. However, no meta-analysis has yet synthesized the evidence specifically within the conventional anthracycline-based prevention framework using small-sample-adjusted inference and absolute mean differences interpretable against the ESC-recommended GLS thresholds. The present study addresses this gap by restricting inclusion to RCTs conducted within this clinically homogeneous setting, applying conservative HKSJ-adjusted inference, and prespecifying final GLS as the primary outcome.

## 2. Materials and Methods

### 2.1. Protocol and Registration

This systematic review and meta-analysis was conducted in accordance with the Preferred Reporting Items for Systematic Reviews and Meta-Analyses (PRISMA) 2020 guidelines [23]. The protocol was registered prospectively in PROSPERO (registration number CRD420261383124).

### 2.2. Eligibility Criteria

Studies were eligible if they were RCTs that (1) enrolled patients receiving anthracycline-containing cancer therapy (with or without trastuzumab, taxanes, or radiation) without pre-existing left ventricular systolic dysfunction (LVEF ≥50%) and without established heart failure; (2) compared sacubitril/valsartan to placebo or standard care; (3) reported GLS as a primary or secondary outcome; and (4) had a minimum follow-up of 6 months. Exclusion criteria were non-randomized studies, retrospective analyses, studies of sacubitril/valsartan for treatment of established CTRCD with reduced LVEF or symptomatic heart failure, and studies not reporting GLS data.

### 2.3. Information Sources and Search Strategy

A systematic search was performed on February 1, 2026, across three electronic databases: PubMed/MEDLINE (*n* = 1858 records), EMBASE via Ovid (*n* = 256), and Cochrane CENTRAL (*n* = 33). The search strategy combined MeSH terms and free-text keywords, including “sacubitril”, “valsartan”, “LCZ696”, “angiotensin receptor neprilysin inhibitor”, “cardiotoxicity”, “cancer therapy-related cardiac dysfunction”, “global longitudinal strain”, “anthracycline”, and “prevention”. Reference lists of included studies and relevant review articles were manually screened. ClinicalTrials.gov and the International Clinical Trials Registry Platform were searched for ongoing or completed trials.

### 2.4. Study Selection and Data Extraction

Two reviewers independently screened titles, abstracts, and full-text articles against the eligibility criteria. Discrepancies were resolved by consensus. Data were extracted using a standardized form capturing study design, sample size, patient characteristics, intervention details, comparator, outcome measures, and safety endpoints. All three trials reported GLS as negative percentage values; the between-group mean difference was computed as sacubitril/valsartan minus control, such that a negative MD indicates greater GLS preservation in the intervention group. For SARAH, which reported final GLS as medians with 95% confidence intervals, the medians were treated as approximate means given the per-arm sample size (*n* = 56) and the approximate symmetry of the distributions. Where trials reported means or medians with 95% confidence intervals rather than standard deviations (SARAH and PRADA II), per-arm standard deviations were back-calculated from the reported 95% confidence intervals as SD = √*n* × (upper − lower)/(2 × 1.96), following the Cochrane Handbook [24].

### 2.5. Risk of Bias Assessment

Risk of bias was assessed using the Cochrane Risk of Bias 2 (RoB 2) tool for randomized trials [25]. Two reviewers independently assessed each trial across the five RoB 2 domains; discrepancies were resolved by consensus.

### 2.6. Statistical Analysis

All analyses were conducted in R version 4.5.2 (R Foundation for Statistical Computing, Vienna, Austria) within the RStudio integrated development environment (version 2026.1.0.392), using the metafor package (version 4.8-0) [26,27]. Random-effects meta-analysis was performed using restricted maximum likelihood (REML) estimation. The HKSJ adjustment was applied to provide conservative confidence intervals based on the t-distribution rather than the standard normal distribution [28]. Heterogeneity was assessed using the I^2^ statistic and Cochran’s Q test. For the hypotension analysis, a continuity correction of 0.5 was applied to cells with zero events. Given k = 3 studies, formal publication bias assessment with funnel plots was not performed [29]. The certainty of evidence was assessed using the GRADE framework [30].

## 3. Results

### 3.1. Study Selection

The search identified 2147 studies across three databases. After removal of 114 duplicates, 2033 unique records were screened by title and abstract. Five full-text articles were assessed for eligibility. Two were excluded: one for initiating the intervention after completion of chemotherapy in the peri-transplant period, and one for enrolling patients with established cardiac dysfunction and reduced LVEF. Three RCTs met all inclusion criteria and were included in the meta-analysis (Figure S1).

### 3.2. Study Characteristics

The three included RCTs enrolled a total of 350 patients (145 sacubitril/valsartan, 205 controls) (Table 1). Hsu et al. [20] was a single-center, open-label, blinded-endpoint trial conducted in Taiwan that randomized 100 patients with treatment-naïve breast cancer at 1:4 to low-dose sacubitril/valsartan (target 24.5/25.5 mg twice daily) or standard care for 12 months. PRADA II [21] was a multicenter, double-blind, placebo-controlled trial conducted in Norway that randomized 138 women with early breast cancer at 1:1 to full-dose sacubitril/valsartan (target 97/103 mg twice daily) or placebo for 18 months. The SARAH trial [22] was a single-center, double-blind, placebo-controlled trial conducted in Brazil that randomized 114 patients with elevated cardiac troponin I during anthracycline therapy at 1:1 to full-dose sacubitril/valsartan or placebo for 6 months (112 included in the efficacy analysis after 2 oncologic deaths).

### 3.3. Primary Outcome: Final GLS

**Figure 1:**
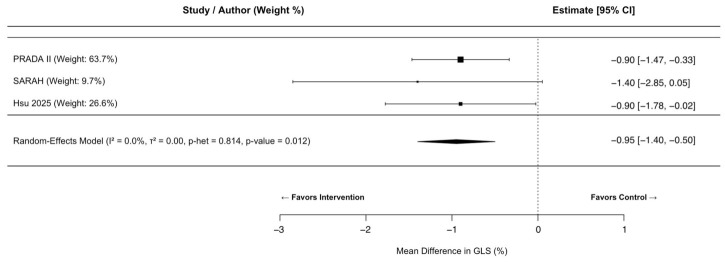
Forest plot showing the pooled between-group mean difference (MD) in final global longitudinal strain (GLS) values between sacubitril/valsartan and control groups across three randomized controlled trials (*n* = 350). Effect sizes are expressed as mean differences (%) with 95% confidence intervals (CIs). The pooled estimate was calculated using a random-effects model with restricted maximum likelihood (REML) estimation and Hartung–Knapp–Sidik–Jonkman (HKSJ) adjustment for small-sample inference. Each square represents the point estimate of an individual trial, with its size proportional to the trial’s weight in the meta-analysis, and the horizontal lines denote the 95% CIs; the diamond represents the overall pooled effect, with its width indicating the 95% CI. The dashed vertical line indicates the line of no effect (MD = 0). More negative values indicate greater GLS preservation, favoring sacubitril/valsartan. The pooled MD was −0.95% (95% CI −1.40 to −0.50; *p* = 0.012; I^2^ = 0%; τ^2^ = 0; Q = 0.41, *p* for heterogeneity = 0.81). Included trials: Hsu et al. [20], PRADA II [21], and SARAH [22].

The pooled between-group MD in final GLS significantly favored sacubitril/valsartan (MD −0.95%; 95% CI −1.40 to −0.50; *p* = 0.012; I^2^ = 0%; τ^2^ = 0; Q = 0.41, *p* for heterogeneity = 0.81) (Figure 1). In the convention used for GLS (where more negative values indicate better function), a negative MD indicates superior GLS preservation in the sacubitril/valsartan group. Study-level weights were: PRADA II 63.7%, Hsu 26.6%, and SARAH 9.7%. The absence of statistical heterogeneity (I^2^ = 0%) indicates a consistent treatment effect across all three trials.

A leave-one-out analysis showed that the pooled MD for final GLS remained negative and of similar magnitude when any single trial was omitted (range −0.90% to −1.03%; Table S1), with no single trial driving the result. The iteration omitting SARAH, the only trial contributing data converted from reported medians, yielded a pooled MD of −0.90%, consistent with the primary estimate. Because each iteration retained two trials, HKSJ inference was based on a single degree of freedom, widening the confidence intervals independently of the effect size; the point estimates are therefore the informative output of this analysis (Table S1).

### 3.4. Sensitivity Analysis: Change-from-Baseline GLS

The sensitivity analysis using change-from-baseline GLS (ΔGLS) showed a pooled MD of −0.92% (95% CI −2.94 to 1.11; *p* = 0.19; I^2^ = 64.2%; τ^2^ = 0.38) (Figure S2). The direction of effect was consistent with the primary analysis, favoring sacubitril/valsartan, but did not reach statistical significance. The moderate heterogeneity likely reflects clinical differences in populations, timing of intervention, and follow-up duration.

### 3.5. Secondary Outcome: Final LVEF

**Figure 2:**
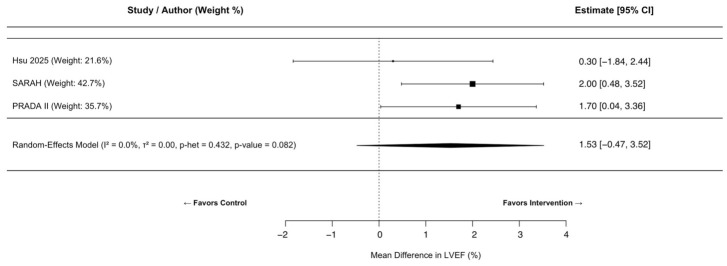
Forest plot showing the pooled between-group mean difference (MD) in final left ventricular ejection fraction (LVEF) values between sacubitril/valsartan and control groups across three randomized controlled trials (*n* = 350). Effect sizes are expressed as mean differences (%) with 95% confidence intervals (CIs). The pooled estimate was calculated using a random-effects model with restricted maximum likelihood (REML) estimation and Hartung–Knapp–Sidik–Jonkman (HKSJ) adjustment for small-sample inference. Each square represents the point estimate of an individual trial, with its size proportional to the trial’s weight in the meta-analysis, and the horizontal lines denote the 95% CIs; the diamond represents the overall pooled effect, with its width indicating the 95% CI. The dashed vertical line indicates the line of no effect (MD = 0). Positive values indicate higher LVEF, favoring sacubitril/valsartan. The pooled MD was 1.53% (95% CI −0.47 to 3.52; *p* = 0.082; I^2^ = 0.0%; τ^2^ = 0.00; *p* for heterogeneity = 0.432). Included trials: Hsu et al. [20], PRADA II [21], and SARAH [22].

The pooled between-group MD in final LVEF favored sacubitril/valsartan numerically but did not reach statistical significance (MD +1.53 percentage points; 95% CI −0.47 to 3.52; *p* = 0.082; I^2^ = 0%; Q = 1.68, *p* for heterogeneity = 0.432) (Figure 2). LVEF was assessed by cardiac magnetic resonance in PRADA II and by echocardiography in Hsu and SARAH.

### 3.6. Safety Outcome: Hypotension

Hypotension was numerically more frequent in the sacubitril/valsartan group, though this did not reach statistical significance after HKSJ adjustment (OR 5.10; 95% CI 0.52–49.50; *p* = 0.091; I^2^ = 4%) (Figure S3). Event rates were: Hsu 2/20 (10%) vs. 0/80 (0%), PRADA II 8/69 (11.6%) vs. 3/69 (4.3%), and SARAH 8/57 (14.0%) vs. 1/57 (1.8%). Across all three trials, no serious adverse events directly attributable to hypotension were reported, and sacubitril/valsartan did not lead to discontinuation of planned cancer therapy.

### 3.7. Exploratory Analysis: Incidence of Clinically Significant GLS Decline

Two trials reported the incidence of clinically significant GLS decline (≥15% relative reduction from baseline) as a standalone dichotomous outcome; PRADA II reported this outcome only as part of a composite endpoint that included both GLS decline and biomarker elevation, precluding extraction of isolated GLS decline data. In Hsu, no patient in the sacubitril/valsartan group developed a ≥15% GLS decline compared with 21 of 80 (26.3%) in the standard care group (*p* = 0.006). In SARAH, a ≥15% GLS decline occurred in 4 of 56 (7.1%) sacubitril/valsartan patients compared with 14 of 56 (25.0%) placebo patients (OR 0.23; 95% CI 0.07–0.75; *p* = 0.015) [22]. Meta-analysis was not performed for this exploratory outcome, given data availability from only two trials.

### 3.8. Risk of Bias

PRADA II and SARAH were judged to have low overall risk of bias across all five RoB 2 domains. Hsu et al. was judged to have some concerns due to its open-label design and unequal 1:4 randomization resulting in a small intervention group (*n* = 20).

### 3.9. Certainty of Evidence

The GRADE assessment is presented in Table 2. For the primary outcome (final GLS), the certainty of evidence was rated as moderate, downgraded one level for risk of bias (one of three trials used an open-label design with unequal randomization). For the secondary outcome (final LVEF), the certainty was rated as very low, downgraded for risk of bias, indirectness (different imaging modalities across trials: CMR in PRADA II, echocardiography in Hsu and SARAH), and imprecision (confidence interval crossing the null). For the sensitivity analysis (change-from-baseline GLS), the certainty was rated as very low, downgraded for risk of bias, inconsistency (I^2^ = 64.2%), and imprecision. For the safety outcome (hypotension), the certainty was rated as low, downgraded for risk of bias and imprecision.

## 4. Discussion

This systematic review and meta-analysis provides pooled quantitative evidence that sacubitril/valsartan preserves global longitudinal strain (GLS) in patients receiving potentially cardiotoxic anthracycline-based cancer therapy. Restricted to randomized controlled trials within the conventional CTRCD prevention spectrum, the pooled analysis of three RCTs (*n* = 350) demonstrated a statistically significant preservation of final GLS (MD −0.95%; *p* = 0.012) with no heterogeneity (I^2^ = 0%), suggesting a consistent cardioprotective signal across trials despite substantial differences in patient selection, dosing, and follow-up duration.

### 4.1. Comparison with Concurrent Meta-Analyses

Two concurrent meta-analyses—Wu et al. [31] and Huntermann et al. [32]—both pooled four RCTs, including the Katogiannis trial [33] involving patients undergoing bone marrow transplantation (BMT) in which anthracycline-based chemotherapy had already been completed before randomization. The primary cardiotoxic insult in the BMT setting is the conditioning regimen itself, representing a fundamentally different clinical question from prevention during active anthracycline exposure. Notably, in Wu et al., sensitivity analysis excluding the Katogiannis trial attenuated the GLS effect to non-significance (SMD 0.40; 95% CI −0.19 to 0.99; *p* = 0.099), confirming that the pooled result in that analysis depended critically on inclusion of this mechanistically heterogeneous population. Furthermore, neither analysis applied the HKSJ adjustment for small-sample inference, and Wu et al. analyzed GLS as a standardized mean difference (SMD) rather than absolute MD, reducing clinical interpretability relative to the ESC-recommended threshold. The present analysis addresses all three limitations.

### 4.2. Comparison with Prior Cardioprotective Strategies

Multiple RCTs of conventional neurohormonal blockers—including candesartan (PRADA) [10], carvedilol (CECCY) [11], combined carvedilol and candesartan (Cardiac CARE) [12], and enalapril or carvedilol (PROACT) [13]—have demonstrated modest or no sustained cardioprotective effects. A meta-analysis of ACE inhibitors and ARBs for primary prevention of CTRCD found no significant reduction in cardiotoxicity events [34]. The potential mechanistic advantage of sacubitril/valsartan over single-pathway inhibitors is supported by preclinical data demonstrating attenuation of systolic dysfunction, reduction of myocardial fibrosis, and promotion of autophagy through regulation of the AMPKα-mTORC1 pathway [18,19].

### 4.3. Clinical Significance and Heterogeneity in Patient Selection

The pooled MD of −0.95% represents a modest absolute difference in terms of absolute GLS units; however, when expressed as a proportion of the mean baseline GLS across all three trials (~19.3%), this corresponds to an approximate relative preservation of 4.9%. The 2022 ESC cardio-oncology guidelines define subclinical CTRCD as a relative GLS decline of ≥15% from baseline; the observed 5% relative preservation thus represents approximately one-third of this diagnostic threshold. Several additional points support its potential clinical relevance. First, GLS decline during chemotherapy has strong prognostic value for subsequent CTRCD [5]. Second, early detection and treatment of subclinical cardiotoxicity are critical for cardiac function recovery [4]. Third, the consistency of the effect across all three trials (I^2^ = 0%) despite their clinical heterogeneity—including differences in patient selection (unselected vs. troponin-positive), dosing strategies (low vs. full dose), cancer types, and concurrent cardiotoxic therapies (trastuzumab in 5–40%, taxanes in 62–65% of PRADA II, radiation in 72–83%)—strengthens the case that the observed GLS preservation reflects a genuine biological signal. An important observation is that SARAH—which specifically enrolled patients with elevated troponin I during anthracycline therapy, representing a biomarker-enriched high-risk population—demonstrated the most pronounced directional effect on GLS preservation among the three trials, despite contributing only 9.7% of the pooled weight. This pattern raises the possibility that the cardioprotective effect of sacubitril/valsartan may be amplified in patients with evidence of early myocardial injury, and that a biomarker-guided strategy—identifying high-risk patients through troponin surveillance and initiating ARNI in those with early biochemical evidence of cardiotoxicity—might improve the benefit-to-risk ratio of this intervention. This hypothesis is consistent with the broader principle of risk-stratified cardioprotection endorsed by the 2022 ESC cardio-oncology guidelines, but requires confirmation in future adequately powered prospective studies.

### 4.4. Rationale for the Primary Outcome Metric

Final GLS rather than change-from-baseline GLS was prespecified as the primary outcome (PROSPERO CRD420261383124), for both statistical and clinical reasons. Because randomization produces expected baseline balance between arms, a comparison of final between-group means provides an unbiased estimate of the treatment effect. The choice also avoids two limitations of the change-from-baseline metric. First, contrary to common assumption, a change score does not correct for baseline imbalance; it is negatively associated with the baseline value and is therefore subject to regression to the mean, whereas a comparison of final values under randomization is not [35]. Second, the relative efficiency of change-score versus final-value analysis depends on the pre–post correlation; when this correlation is not high, a final-value analysis is at least as powerful [35,36]. Although covariate-adjusted (ANCOVA) estimates would be the most efficient approach, these cannot be reconstructed from published summary-level data; among the two approaches recoverable from the trial reports, final values were therefore preferred. This is consistent with the heterogeneity we observed: the final-GLS analysis was homogeneous (I^2^ = 0%), whereas the change-from-baseline analysis was heterogeneous (I^2^ = 64.2%), reflecting genuine clinical differences across trials (troponin-positive versus unselected populations, dosing, and follow-up duration). The change-from-baseline analysis was nonetheless retained as a supportive sensitivity analysis because the 2022 ESC threshold for subclinical CTRCD is defined as a relative GLS decline (≥15% from baseline), a change-based metric. The direction and magnitude of effect were concordant across both approaches (MD −0.95% for final GLS versus MD −0.92% for ΔGLS), which supports the finding, while the non-significance of the change-from-baseline estimate tempers its interpretation and reinforces the need for confirmation in adequately powered trials.

### 4.5. LVEF vs. GLS as Outcome Measure

The present meta-analysis provides direct evidence of the differential sensitivity of GLS vs. LVEF: sacubitril/valsartan was associated with significant GLS preservation (*p* = 0.012) but not with significant LVEF improvement (*p* = 0.082), despite both analyses showing zero heterogeneity and a consistent direction of effect. This discordance is likely explained by the fact that with contemporary low-to-moderate dose anthracycline regimens, LVEF decline in the control arms was modest (1–2 percentage points), falling within the measurement variability of echocardiography. These findings support the 2022 ESC guidelines’ recommendation of GLS-based surveillance during cancer therapy [7].

### 4.6. Safety Considerations

Hypotension was the most notable safety signal, with a numerically higher odds in the sacubitril/valsartan group that did not reach statistical significance after conservative HKSJ adjustment (OR 5.10; *p* = 0.091). This finding is clinically relevant given that oncology patients may experience hemodynamic instability from chemotherapy-related dehydration and myelosuppression. However, across all three trials, hypotensive episodes were generally manageable with dose reduction and sacubitril/valsartan did not lead to interruption of cancer therapy. The wide confidence interval around this estimate (95% CI 0.52–49.50) reflects the small number of hypotensive events and the limited sample size, so the magnitude of this risk remains uncertain; the consistent direction across trials nonetheless suggests a possible safety signal that warrants evaluation in adequately powered studies.

### 4.7. Future Directions

The ongoing MAINSTREAM trial (NCT05465031) is a multicenter, randomized, double-blind, placebo-controlled trial evaluating sacubitril/valsartan in 480 patients with breast cancer undergoing anthracycline-based chemotherapy, with a 24-month follow-up and LVEF as the primary endpoint [37]. This trial is expected to provide definitive evidence regarding whether the GLS preservation demonstrated in the current meta-analysis translates into clinical prevention of cardiac dysfunction. Additional outcomes of interest—including biomarker changes, clinical heart failure events, and optimal patient selection (universal vs. biomarker-guided)—could not be reliably pooled given the small number of studies and heterogeneous reporting, and warrant prospective evaluation. Future studies should also evaluate the potential synergistic effects of combining ARNI with other emerging cardioprotective strategies such as sodium-glucose cotransporter 2 inhibitors, which have shown promise in attenuating cardiotoxicity in patients undergoing chemotherapy [38].

### 4.8. Limitations

Several limitations should be acknowledged. First, only three RCTs were available for inclusion, limiting statistical power and precluding formal assessment of publication bias; the possibility of unpublished neutral or negative trials cannot be excluded, and small meta-analyses such as this are susceptible to it, which could attenuate the pooled effect if such data existed. The limited number of trials also precluded quantitative subgroup analyses (e.g., by sacubitril/valsartan dose, baseline troponin status, concurrent trastuzumab or thoracic radiation, or tumor type), which would require a larger evidence base. Second, there was clinical heterogeneity across trials in patient selection criteria, sacubitril/valsartan dosing (low- vs. target-dose), study design (open-label vs. double-blind), follow-up duration (6 to 18 months), and concomitant oncologic therapies. Third, pooling of final values across differing follow-up durations assumes that treatment effects are adequately captured at the longest available time point, which may not fully reflect temporal dynamics of cardiotoxicity. Fourth, the SARAH trial reported outcomes as medians with 95% confidence intervals, requiring conversion to means and standard deviations; however, a leave-one-out analysis excluding SARAH yielded a consistent pooled estimate (MD −0.90%; Table S1), indicating that this approximation did not alter the primary result. Fifth, the Hsu study employed unequal randomization (1:4), resulting in a relatively small intervention group (*n* = 20), which may affect precision. Sixth, the LVEF analysis required the use of baseline standard deviations as proxies for final values in one study and combined measurements obtained using different imaging modalities (cardiac magnetic resonance in PRADA II vs. echocardiography in the other trials), introducing potential measurement variability. Seventh, although GLS is a validated imaging biomarker incorporated into guideline-based definitions of CTRCD [7], it remains a surrogate endpoint; whether preservation of GLS translates into reductions in clinically overt heart failure or mortality requires confirmation in larger trials with hard clinical outcomes. These findings apply specifically to primary prevention initiated during active anthracycline-based therapy and do not extend to the treatment of established subclinical or overt CTRCD, a distinct clinical indication in which sacubitril/valsartan has been evaluated in a retrospective registry [39]. Finally, the pooled population consisted predominantly of women with breast cancer; non-breast malignancies (lymphoma, sarcoma, and leukemia) were present in only one trial, and men made up a small minority of the cohort. The applicability of these findings to men or to patients with other malignancies is therefore uncertain.

## 5. Conclusions

In this meta-analysis of three randomized controlled trials (*n* = 350) within the anthracycline-based CTRCD prevention setting, sacubitril/valsartan was associated with a statistically significant but modest preservation of final GLS (MD −0.95%; 95% CI −1.40 to −0.50), without a significant effect on LVEF. The direction of effect was concordant across analyses, although the change-from-baseline sensitivity analysis did not reach significance, and a numerically higher rate of hypotension was observed. These findings should be interpreted with caution: GLS is a surrogate imaging marker; clinical events (heart failure, hospitalization, and death) were secondary or exploratory endpoints that were defined and reported inconsistently across trials and were rare or absent, so a quantitative synthesis of hard clinical outcomes was not feasible and preserved GLS cannot yet be equated with a reduction in clinical events. While the meta-analysis provides consistent randomized evidence for the attenuation of subclinical myocardial injury, its translation into clinical benefit remains uncertain. Adequately powered, multicenter trials with longer follow-up and prespecified clinical endpoints are needed before sacubitril/valsartan can be recommended for cardioprotection during cancer therapy.

## Figures and Tables

**Table 1:** Baseline Characteristics of Included Randomized Controlled Trials.

Characteristic	Hsu et al., 2025 Eur J Heart Fail [20]	PRADA II, 2025 Circulation [21]	SARAH, 2025 Circulation [22]
**Study Design**			
Country	Taiwan	Norway (multicenter)	Brazil
Design	Open-label, blinded-endpoint	Double-blind, placebo-controlled	Double-blind, placebo-controlled
Randomization ratio	1:4 (ARNI:control)	1:1	1:1
Sacubitril/valsartan target dose	24.5/25.5 mg BID (low-dose)	97/103 mg BID	97/103 mg BID
Follow-up duration	12 months	18 months	6 months
Troponin-guided enrollment	No	No	Yes (hs-cTnI > 99th percentile)
**Patient Characteristics**			
Total *n* randomized	100	138	114
ARNI group, n	20	69	57
Control group, n	80	69	57
Age, years, mean ± SD	50.1 ± 6.1 vs. 51.5 ± 9.0	53.6 ± 9.2 vs. 54.5 ± 9.7	51.6 ± 11.6 vs. 51.7 ± 11.6
Female sex, %	100 vs. 97.5	100 vs. 100	93.0 vs. 87.7
Breast cancer, %	100 vs. 100	100 vs. 100	82.5 vs. 79.0
Lymphoma, %	—	—	15.7 vs. 17.4
Sarcoma, %	—	—	1.8 vs. 1.8
Hypertension, %	15 vs. 20	Not reported separately	31.6 vs. 31.6
Diabetes, %	0 vs. 10	4.3 vs. 1.4	10.5 vs. 8.8
**Oncologic Therapy**			
Anthracycline regimen	Doxorubicin-based	Epirubicin-cyclophosphamide	Doxorubicin-based
Anthracycline use, %	90 vs. 85	100 vs. 100	100 vs. 100
Anthracycline dose, doxorubicin equivalent mg/m^2^	276.0 ± 48.5 vs. 269.5 ± 57.9	EC 90 mg/m^2^ × 4 (81.2% vs. 88.4% received planned dose) ^a^	240 (236–242) vs. 239 (231–240) ^1^
Trastuzumab, %	5 vs. 22.5	17.4 vs. 15.9	19.1 vs. 40.0 ^2^
Radiotherapy, %	Not reported	82.6 vs. 79.7	71.9 vs. 71.9
**Baseline Cardiac Parameters**			
Primary imaging modality	Echocardiography	Cardiac MRI (CMR)	Echocardiography + CMR
Baseline LVEF, %, mean ± SD	63.1 ± 3.7 vs. 63.4 ± 4.1	60.6 (59.4–61.7) vs. 60.0 (58.8–61.1) ^3^	64.4 ± 4.9 vs. 63.7 ± 3.9
Baseline GLS, %, mean ± SD	−20.4 ± 1.9 vs. −19.7 ± 1.9	−19.1 (−19.5 to −18.7) vs. −19.0 (−19.4 to −18.6) ^3^	−20.2 (−22.4, −18.0) vs. −20.1 (−21.9, −18.7) ^2^
Baseline hs-cTnI, ng/L	4.8 ± 1.3 vs. 5.7 ± 8.4 (hs-cTnT)	Low (all below detection limit)	24.9 (16.9–37.9) vs. 19.1 (13.0–35.3) ^1,4^
Baseline NT-proBNP, pg/mL	60.8 ± 29.6 vs. 56.9 ± 59.0	Normal (specific values not reported at baseline)	10 (4–27) vs. 12 (5–25) ^1^

^1^ Values expressed as median (25th–75th percentile) for SARAH, as GLS and most continuous variables were non-normally distributed. ^2^ Imbalance in trastuzumab use between groups in SARAH (19.1% ARNI vs. 40.0% placebo) was not statistically significant and did not significantly affect the primary outcome in sensitivity analyses. ^3^ Values expressed as mean (95% CI) for PRADA II, as reported in the primary publication. ^4^ Baseline hs-cTnI in SARAH refers to values at the prerandomization time point (T2, after anthracycline exposure triggering troponin elevation above the 99th percentile), not at the pre-chemotherapy baseline (T1). ^a^ PRADA II used epirubicin–cyclophosphamide (EC 90 mg/m^2^ ×4 cycles); mean doxorubicin-equivalent dose was not reported in the primary publication. Values represent the proportion of patients who received the planned standard regimen in the sacubitril/valsartan and placebo groups. Patients with reduced doses (18.8% vs. 11.6%) received epirubicin–cyclophosphamide at 60 mg/m^2^. ARNI = angiotensin receptor–neprilysin inhibitor; BID = twice daily; CMR = cardiac magnetic resonance; EC = epirubicin–cyclophosphamide; GLS = global longitudinal strain; hs-cTnI = high-sensitivity cardiac troponin I; LVEF = left ventricular ejection fraction; NT-proBNP = N-terminal pro-B-type natriuretic peptide; SD = standard deviation. Unless otherwise indicated, values are expressed as mean ± SD for continuous variables, and percentages for categorical variables, presented as ARNI group vs. control group.

**Table 2:** Certainty of Evidence Assessment Using the GRADE Framework.

Certainty Assessment							No. of Patients		Effect	Certainty
Outcome	Studies (k)	Study Design	Risk of Bias	Inconsistency	Indirectness	Imprecision	ARNI (n)	Control (n)	Absolute Effect (95% CI)	Certainty
Efficacy Outcomes										
GLS, final (primary outcome)	3	RCTs	Serious ^1^	Not serious	Not serious	Not serious	145	205	MD −0.95% (95% CI −1.40 to −0.50) *p* = 0.012; I^2^ = 0%	⊕⊕⊕○ Moderate
LVEF, final (secondary)	3	RCTs	Serious ^1^	Not serious	Serious ^2^	Serious ^3^	145	205	MD +1.53 pp (95% CI −0.47 to +3.52) *p* = 0.082; I^2^ = 0%	⊕○○○ Very low
Sensitivity Analysis										
ΔGLS, change from baseline (sensitivity)	3	RCTs	Serious ^1^	Serious ^4^	Not serious	Serious ^3^	145	205	MD −0.92% (95% CI −2.94 to +1.11) *p* = 0.191; I^2^ = 64.2%	⊕○○○ Very low
Safety Outcome										
Hypotension (safety)	3	RCTs	Serious ^1^	Not serious	Not serious	Serious ^3^	146	206	OR 5.10 (95% CI 0.52 to 49.50) *p* = 0.091; I^2^ = 4%	⊕⊕○○ Low

^1^ Risk of bias: downgraded one level. The Hsu et al. trial used an open-label, blinded-endpoint design with unequal 1:4 randomization; PRADA II and SARAH were double-blind, placebo-controlled, with low overall risk of bias. ^2^ Indirectness (LVEF only): downgraded one level. LVEF was assessed by different imaging modalities across trials (cardiac magnetic resonance in PRADA II; echocardiography in Hsu and SARAH), introducing potential measurement heterogeneity. ^3^ Imprecision: downgraded one level. The 95% confidence interval crosses the null (for LVEF and ΔGLS) or is very wide (for hypotension, OR 0.52–49.50), reflecting limited precision with k = 3 studies and HKSJ-adjusted inference (t-distribution, df = 2). ^4^ Inconsistency (ΔGLS only): downgraded one level. I^2^ = 64.2% indicates moderate heterogeneity, likely reflecting differences in patient selection (unselected vs. troponin-positive), follow-up duration (6–18 months), and sacubitril/valsartan dosing strategy across trials. Abbreviations: ΔGLS = change from baseline GLS; CI = confidence interval; CMR = cardiac magnetic resonance; GLS = global longitudinal strain; HKSJ = Hartung–Knapp–Sidik–Jonkman; LVEF = left ventricular ejection fraction; MD = mean difference; OR = odds ratio; pp = percentage points; RCT = randomized controlled trial. GRADE certainty symbols: |⊕⊕⊕○ Moderate|⊕⊕○○ Low|⊕○○○ Very Low.

## Data Availability

All data generated or analyzed during this study are included in this published article and its Supplementary Materials. The data extraction forms and statistical analysis code are available from the corresponding author upon reasonable request.

## Supplementary Materials

The following supporting information can be downloaded at: https://www.mdpi.com/article/10.3390/jcdd13070323/s1.

## Abbreviations

The following abbreviations are used in this manuscript:

ACE	Angiotensin-converting enzyme
ARB	Angiotensin receptor blocker
ARNI	Angiotensin receptor–neprilysin inhibitor
BMT	Bone marrow transplantation
CI	Confidence interval
CMR	Cardiac magnetic resonance
CTRCD	Cancer therapy-related cardiac dysfunction
ESC	European Society of Cardiology
GLS	Global longitudinal strain
GRADE	Grading of Recommendations Assessment, Development and Evaluation
HER2	Human epidermal growth factor receptor 2
HKSJ	Hartung–Knapp–Sidik–Jonkman
LVEF	Left ventricular ejection fraction
MD	Mean difference
NT-proBNP	N-terminal pro-B-type natriuretic peptide
OR	Odds ratio
PRISMA	Preferred Reporting Items for Systematic Reviews and Meta-Analyses
RCT	Randomized controlled trial
REML	Restricted maximum likelihood
RoB	Risk of bias
SD	Standard deviation
