# Supplementary material for: Sacubitril/Valsartan for Prevention of Cancer Therapy-Related Cardiac Dysfunction: A Systematic Review and Meta-Analysis of Randomized Controlled Trials
Source: J Cardiovasc Dev Dis. 2026 Jul 10;13(7):323. doi: 10.3390/jcdd13070323 (PMC13410051; doi:10.3390/jcdd13070323)
Supplement: Supplementary file 1 [file jcdd-13-00323-s001.zip › jcdd-4353943-supplementary.pdf]

Supplementary Materials

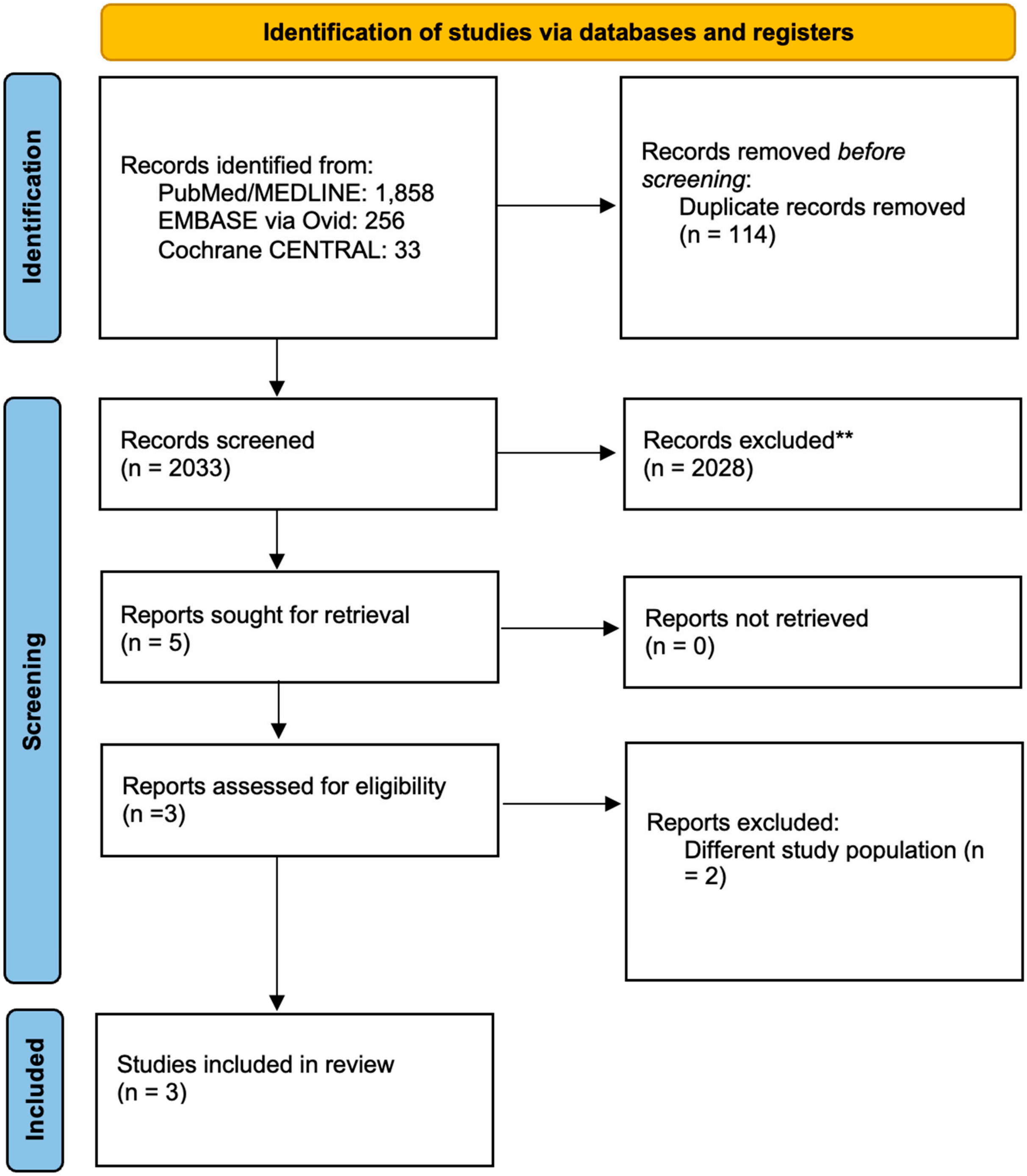

Figure S1 PRISMA 2020 flow diagram of study selection for the systematic review and meta-analysis.

Source: Page MJ, et al. BMJ 2021;372:n71. doi: 10.1136/bmj.n71.

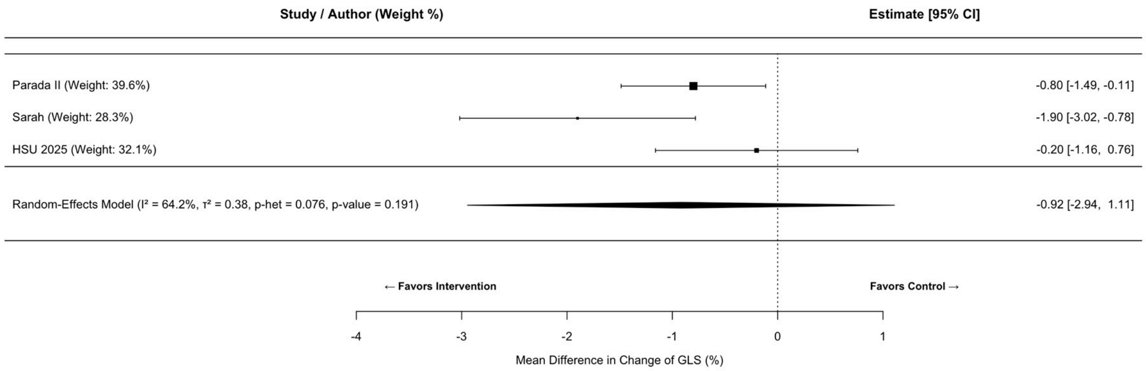

Figure S2. Forest plot showing the pooled between-group mean difference (MD) in the longitudinal change of global longitudinal strain (GLS) from baseline to follow-up between sacubitril/valsartan and control groups (N=350). Effect sizes are expressed as mean differences (%) with 95% confidence intervals (CIs). The pooled estimate was calculated using a random-effects model with restricted maximum likelihood (REML) estimation and Hartung–Knapp–Sidik–Jonkman (HKSJ) adjustment for small-sample inference. The diamond represents the overall pooled effect. More negative values indicate greater GLS preservation, favoring sacubitril/valsartan. The pooled MD was −0.92% (95% CI −2.94 to 1.11; p=0.191; I^2=64.2%; τ^2=0.38; p for heterogeneity=0.076).

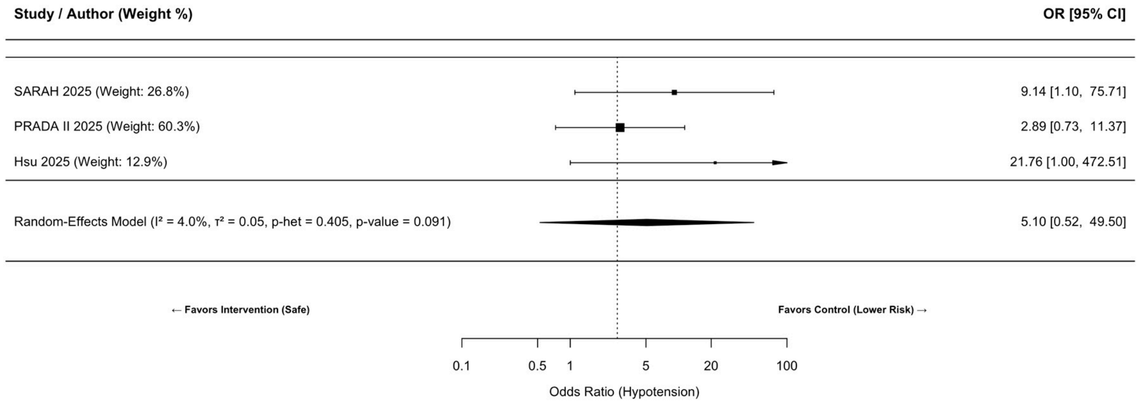

Figure S3. Forest plot showing the pooled between-group Odds Ratio (OR) for hypotension between sacubitril/valsartan and control groups across three randomized controlled trials (N=352). Effect sizes are expressed as Odds Ratios with 95% confidence intervals (CIs). The pooled estimate was calculated using a random-effects model with restricted maximum likelihood (REML) estimation and Hartung–Knapp–Sidik–Jonkman (HKSJ) adjustment for small-sample inference. The diamond represents the overall pooled effect. Values greater than 1.0 indicate a higher risk of hypotension in the sacubitril/valsartan group, favoring the control group for this safety endpoint. The pooled OR was 5.10 (95% CI 0.52 to 49.50; p=0.091; I^2=4.0%; τ^2=0.05; p for heterogeneity=0.405).

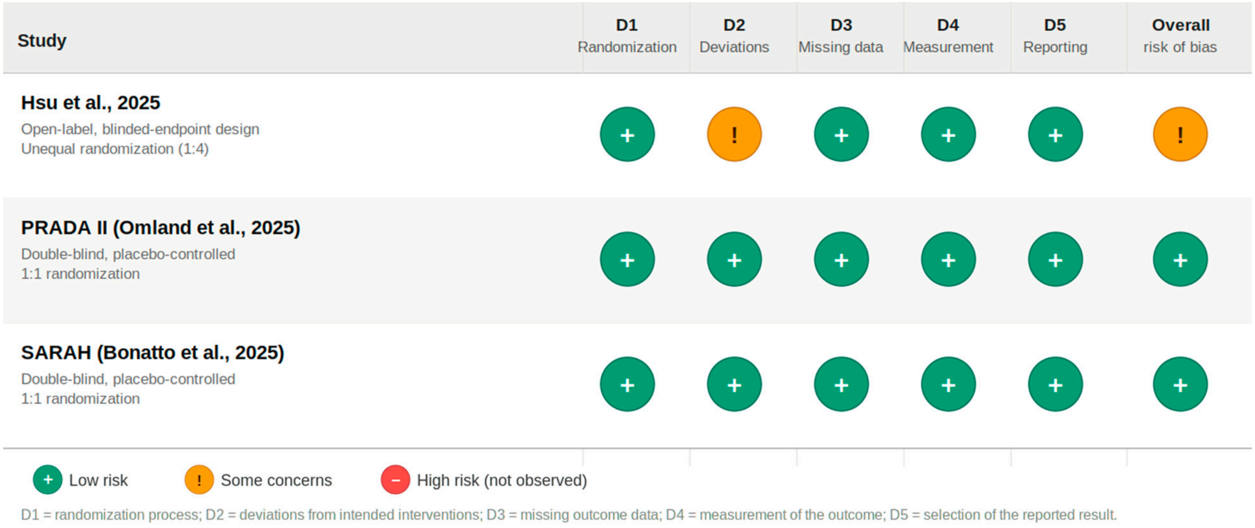

Figure S4. Risk of bias assessment of included randomized controlled trials using the Cochrane Risk of Bias 2 (RoB 2) tool. Each domain was independently evaluated by two reviewers. D1 = randomization process; D2 = deviations from intended interventions; D3 = missing outcome data; D4 = measurement of the outcome; D5 = selection of the reported result. Green circles (+) indicate low risk of bias; yellow circles (!) indicate some concerns. PRADA II (Omland et al., 2025) and SARAH (Bonatto et al., 2025) were double-blind, placebo-controlled trials rated as low overall risk of bias across all five domains. Hsu et al. (2025) was an open-label, blinded-endpoint trial rated as having some concerns in D2 due to absence of participant and investigator blinding, resulting in an overall judgment of some concerns.

Table S1. Leave-one-out influence analysis for the primary outcome (final GLS).

Trial omitted	Studies remaining (k)	Pooled MD (%)	95% CI	I^2 (%)
HSU 2025	2	−0.97	−3.12 to 1.18	0
SARAH	2	−0.90	−0.90 to −0.90 †	0
PRADA II	2	−1.03	−3.84 to 1.78	0
**None (full model)**	**3**	**−0.95**	**−1.40 to −0.50**	**0**

* With k = 2 per iteration, Hartung–Knapp–Sidik–Jonkman inference has 1 degree of freedom; the confidence intervals are valid but uninformative and are shown for transparency only. Significance of the primary outcome is established by the full model (bottom row).

† The confidence interval collapses to a point because the two remaining trials have identical mean differences (−0.9%), eliminating between-study dispersion.

Abbreviations: CI, confidence interval; GLS, global longitudinal strain; HKSJ, Hartung–Knapp–Sidik–Jonkman; MD, mean difference. Random-effects meta-analysis (REML) with HKSJ adjustment.

Search strategy

PubMed/MEDLINE:
("sacubitril and valsartan sodium hydrate drug combination"[Supplementary Concept] OR "sacubitril valsartan" OR LCZ696 OR ARNI) AND ("Heart Failure"[Mesh] OR "Ventricular Dysfunction"[Mesh] OR Cardiotoxicity OR "Heart Failure" OR CTRCD OR "global longitudinal strain" OR GLS OR "ventricular dysfunction") AND (Neoplasms OR Anthracycline OR cancer OR oncology OR chemotherapy OR HER2 OR trastuzumab)

Embase:
('sacubitril and valsartan sodium hydrate drug combination' OR 'sacubitril valsartan' OR 'lcz696' OR 'arni') AND ('heart failure'/exp OR 'heart ventricle function'/exp OR 'cardiotoxicity' OR 'heart failure' OR 'ctrcd' OR 'global longitudinal strain' OR 'gls' OR 'ventricular dysfunction') AND ('neoplasms' OR 'anthracycline' OR 'cancer' OR 'oncology' OR 'chemotherapy' OR 'her2' OR 'trastuzumab')

Cochrane CENTRAL: ("sacubitril valsartan" OR LCZ696 OR ARNI) AND ([mh Cardiotoxicity] OR [mh "Heart Failure"] OR Cardiotoxicity OR "Heart Failure" OR CTRCD OR "global longitudinal strain" OR GLS OR "ventricular dysfunction") AND ([mh Neoplasms] OR cancer OR oncology OR chemotherapy OR [mh Anthracyclines] OR anthracycline OR HER2 OR trastuzumab)
